# *Deinococcus radiodurans* R1 Lysate Induces Tolerogenic Maturation in Lipopolysaccharide-Stimulated Dendritic Cells and Protects Dextran Sulfate Sodium-Induced Colitis in Mice

**DOI:** 10.4014/jmb.2203.03008

**Published:** 2022-06-07

**Authors:** Ha-Yeon Song, Jeong Moo Han, Woo Sik Kim, Ji Hee Lee, Woo Yong Park, Eui-Baek Byun, Eui-Hong Byun

**Affiliations:** 1 Advanced Radiation Technology Institute, Korea Atomic Energy Research Institute, Jeongeup 56212, Republic of Korea; 2 Department of Biotechnology, College of Life Science and Biotechnology, Korea University, Seoul 02841, Republic of Korea; 3 Functional Biomaterial Research Center, Korea Research Institute of Bioscience and Biotechnology, Jeongeup 56212, Republic of Korea; 4 Division of Pathogen Resource Management, Center for Vaccine Development Support, National Institute of Infectious Disease, National Institute of Health (NIH), Korea Disease Control and Prevention Agency, Cheongju, 28160, Republic of Korea; 5 Department of Pharmacology, College of Korean Medicine, Kyung Hee University, Seoul 02447, Republic of Korea; 6 Department of Food Science and Technology, Kongju National University, Yesan, 32439, Republic of Korea

**Keywords:** *Deinococcus radiodurans* R1 lysates, anti-inflammatory activity, tolerogenic dendritic cells, interleukin-10, inflammatory bowel disease

## Abstract

*Deinococcus radiodurans* is an extremophilic bacterium that can thrive in harsh environments. This property can be attributed to its unique metabolites that possess strong antioxidants and other pharmacological properties. To determine the potential of *D. radiodurans* R1 lysate (DeinoLys) as a pharmacological candidate for inflammatory bowel disease (IBD), we investigated the anti-inflammatory activity of DeinoLys in bone marrow-derived dendritic cells (BMDCs) and a colitis mice model. Lipopolysaccharide (LPS)-stimulated BMDCs treated with DeinoLys exhibited alterations in their phenotypic and functional properties by changing into tolerogenic DCs, including strongly inhibited proinflammatory cytokines (TNF-α and IL-12p70) and surface molecule expression and activated DC-induced T cell proliferation/activation with high IL-10 production. These phenotypic and functional changes in BMDCs induced by DeinoLys in the presence of LPS were abrogated by IL-10 neutralization. Furthermore, oral administration of DeinoLys significantly reduced clinical symptoms against dextran sulfate sodium-induced colitis, including body weight loss, disease activity index, histological severity in colon tissue, and lower myeloperoxidase level in mice. Our results establish DeinoLys as a potential anti-inflammatory candidate for IBD therapy.

## Introduction

Extremophilic microbes are organisms that can flourish in harsh environments, such as deserts, salt lakes, arctic ice, and volcanic regions [1]. In recent years, it has been speculated that some unique components contained in extremophiles offer their survival and protective ability against harsh environmental conditions. Based on this, bioactive substances derived from extremophilic bacteria have been proposed as potential pharmaceutical and nutraceutical ingredients [2, 3]. Among various extremophiles, radioresistant extremophiles have drawn wide attention because of their strong survival ability against excessive exposure to radiation, oxidants, and desiccation [4]. *Deinococcus radiodurans* is known as a champion radioresistant bacterium, and its efficient and accurate DNA repair system is robust [5]. Several studies have suggested that *D. radiodurans* extracts exhibit biological activities in experimental models; for example, crude secondary metabolite extracts obtained from *D. radiodurans* exhibit great anticancer activity in human breast cancer cells [6]. Deinoxanthin, a unique carotenoid synthesized by *D. radiodurans*, exhibits strong anticancer activity in various human cancer cell lines, including HepG2 (hepatocellular carcinoma), HT-29 (colon adenocarcinoma), and PC-3 (prostate cancer) cells [7]. Furthermore, exopolysaccharides produced by *D. radiodurans* possess strong antioxidant capacity, which can protect human keratinocytes under oxidative stress conditions [8]. Although many previous studies have focused on the antioxidant activity of *D. radiodurans* and its potential as an antioxidant agent, there is no evidence to support the anti-inflammatory effects of *D. radiodurans*-driven ingredients.

Innate immune cells recognize pathogen invasion and tissue injury, which trigger inflammatory responses to protect against infection and recover from tissue damage [9]. Dendritic cells (DCs) are important innate immune cells that serve as a link between innate and adaptive immunity, presenting antigens to adaptive immune cells and mediating effector cell polarization [10]. Generally, DCs present the processed antigen on both major histocompatibility complex (MHC)-II and MHC-I molecules to naive CD4^+^ and CD8^+^ T cells, respectively, leading to protective immunity against specific pathogens. In contrast, DCs can also induce regulatory functions under inflammatory conditions that facilitate the maintenance of immune homeostasis [11]. Tolerogenic DCs, in particular, can be generated by a variety of chemicals, organic molecules, and cytokines that can induce regulatory T cells by expressing high levels of anti-inflammatory molecules [12]. Therefore, the modulation of DC phenotype and function into tolerogenic DCs is considered an effective therapeutic strategy for various inflammatory diseases, such as inflammatory bowel disease (IBD), rheumatoid arthritis, and multiple sclerosis [13, 14].

In the present study, we investigated whether *D. radiodurans* R1 lysate (DeinoLys) exhibit anti-inflammatory activity by regulating the phenotype and function of tolerogenic DCs in vitro and consequently protect ulcerative colitis symptoms in a mouse model.

## Materials and Methods

### Reagents and Antibodies

Recombinant mouse granulocyte-macrophage colony stimulating factor (rmGM-CSF) and recombinant mouse interleukin (rmIL)-4 were purchased from JW Creagene (Korea). Roswell Park Memorial Institute (RPMI) 1640 medium, fetal bovine serum (FBS), and penicillin/streptomycin were obtained from Gibco (Grand Island, USA). Lipopolysaccharide (LPS) from *Escherichia coli* O111:B4 (ultra-pure grade) was obtained from InvivoGen (USA). V450-conjugated anti-CD11c, fluorescein isothiocyanate (FITC)-conjugated anti-CD80, anti-IL-10, anti-CD4, annexin V/propidium iodine (PI) kit, phycoerythrin (PE)-conjugated anti-CD86, anti-IL-12p70, allophycocyanin (APC)-conjugated tumor necrosis factor (TNF)-α, PE-Cy7-conjugated anti-CD11c, anti-CD8, BV510-conjugated live/dead kit, and enzyme linked immunosorbent assay (ELISA) kits for TNF-α, IL-12p70, IL-10, IFN-γ, IL-2, and IL-5 were purchased from BD biosciences (USA). APC-conjugated anti-MHC-I, PE-Cy7-conjugated anti-MHC-II, anti-CD8, 2-mercaptoethanol, 4-(2-hydroxyethyl)-1-piperazineethanesulfonic acid (HEPES) buffer, Minimum Essential Medium (MEM) non-essential amino acids, and CellTrace Violet Cell Proliferation Kit were purchased from Invitrogen (USA).

### Preparation of Aqueous *D. radiodurans* lysates (DeinoLys)

*D. radiodurans* R1 (ATCC13939) was obtained from the American Type Culture Collection (ATCC) and cultivated in TGY medium (0.5% tryptone, 0.1% glucose, and 0.3% yeast) at 30°C for 24 h. Culture media were purchased from Difco (USA). *D. radiodurans* R1 lysate (DeinoLys) was obtained using a previously established method with slight modifications [15]. Briefly, the cultured *D. radiodurans* R1 in 8 L of medium was centrifuged at 7,000 rpm and 4°C for 20 min, and the pellet was washed twice with phosphate-buffered saline (PBS). Distilled water (4-fold) was added to 27 g of *D. radiodurans* R1 pellet and shake-cultured at 100°C for 15 min. The extracts were centrifuged at 12,000 rpm at 4°C for 20 min, and the supernatant was harvested, filtered, and freeze-dried. The yield of *D. radiodurans* R1 lysate (DeinoLys) was 2.91%. The DeinoLys powder was dissolved in distilled water at a concentration of 5 mg/ml and filtered using a 0.22 μm syringe filter (Corning, USA). Next, the endotoxin in DeinoLys was determined using the Limulus Amebocyte Lysate (LAL) assay kit (Lonza, Switzerland) following the manufacturer’s instructions. The endotoxin content in DeinoLys was < 0.1 EU/ml.

### Culture of Bone Marrow-Derived Dendritic Cells (BMDCs)

Following a previously described procedure [16], BMDCs were collected from 6–8-week-old C57BL/6 female mice (Orient Bio, Inc., Korea). First, complete bone marrow cells were extracted from the femur bone of mice, and red blood cells (RBC) were removed using the RBC lysis buffer (Sigma-Aldrich, USA). On a 100 mm petri dish, the lysed cells were cultured with 10 ml of RPMI 1640 medium supplemented with 10% FBS, 1% penicillin/streptomycin, 20 ng/ml rmGM-CSF, 0.5 ng/ml rmIL-4, 50 μM 2-mercaptoethanol, 1% MEM nonessential amino acids, and 5 mM HEPES (complete DC media) at 37°C in a 5% CO_2_ incubator. On days 3 and 6 of culture, 10 mL of complete DC medium was added. On day 8, BMDCs were isolated using anti-CD11c microbeads (Miltenyi Biotec, Germany) from suspended cells. All experimental procedures were approved by the Institutional Animal Care and Use Committee of the Korea Atomic Energy Research Institute (KAERI-IACUC-2020-005).

### Cell Viability Analysis

BMDCs were seeded at a density of 0.25 × 10^6^ cells per well on a 48-well plate and treated with DeinoLys (100, 200, and 500 μg/ml) in the presence or absence of LPS (100 ng/ml) for 24 h. Cells were co-treated for 24 hours with DeinoLys and LPS in the LPS + DeinoLys group. Cells were harvested and washed with PBS twice and subsequently stained with FITC-conjugated annexin V/PI kit following the manufacturer’s protocol. Flow cytometry (MACSQuant VYB, Miltenyi Biotec) was used to detect the dead cells. Flow cytometry data were analyzed using FlowJo version 10 software (TreeStar, USA).

### Cytokine Measurement in BMDCs

The Extracellular Levels of TNF-α, IL-12p70, and IL-10 in BMDC Culture supernatants were measured using commercial ELISA kits following the manufacturer’s protocol. The intracellular levels of TNF-α, IL-12p70, and IL-10 were determined as previously described [17]. Briefly, BMDCs were treated with DeinoLys (500 μg/ml), LPS (100 ng/ml), or a combination of DeinoLys and LPS in the presence of 1 μg/ml GolgiPlug (BD Biosciences) for 9 h. BMDCs were subsequently stained with PE-Cy7-conjugated anti-CD11c and BV510-conjugated live/dead kits for 30 min at 4°C. The cells were then fixed and permeabilized using a Cytofix/Cytoperm kit (BD Biosciences), and stained with anti-TNF-α, PE-conjugated anti-IL-12p70, and anti-IL-10 mAbs conjugated to APC, PE, and FITC, respectively. The intracellular levels of TNF-α, IL-12p70, and IL-10 in CD11c^+^ cells were analyzed using flow cytometry.

### Surface Molecules Measurement

BMDCs were treated with LPS, DeinoLys, or a combination of DeinoLys and LPS for 24 h and then stained with V450-conjugated anti-CD11c, FITC-conjugated anti-CD80, PE-conjugated anti-CD86, APC-conjugated anti-MHC-I, PE-Cy7-conjugated anti-MHC-II, and BV510-conjugated live/dead kit for 20 min at 25°C. The expression levels of surface molecules in CD11c^+^ cells were analyzed using flow cytometry.

### Mixed Lymphocyte Reaction (MLR) Assay

Following the established methodology, single-cell suspensions of splenocytes were obtained from 7-week-old BALB/c mice (Orient Bio, Inc.) [17]. CD4^+^ and CD8^+^ T cells were separated from splenocytes using anti-CD4 or anti-CD8-coated magnetic microbeads (Miltenyi Biotec) and LS columns (Miltenyi Biotec) according to the manufacturer’s protocol. The isolated CD4^+^ and CD8^+^ T cells were stained with CellTrace Violet Cell Proliferation Kit for 20 min at 37°C and washed with 2% FBS in PBS for 10 min. The CellTrace-labeled CD4^+^ and CD8^+^ T cells were cocultured with each group of BMDCs (C57BL/6 background) for 48 h on 1 μg/ml anti-CD3-coated round-bottom 96-well plates in the presence of 1 μg/ml anti-CD28 (DC:T cell ratio 1:5). After coculturing, the cells were stained with FITC-conjugated anti-CD4 and PE-Cy7-conjugated anti-CD8 mAbs for 20 min at 25°C. The proliferation of CD4^+^ and CD8^+^ T cells was detected using flow cytometry. The levels of IFN-γ, IL-2, and IL-5 in the culture supernatants were measured using commercial ELISA kits.

### Inhibition of IL-10 by Neutralizing Antibody

Anti-mouse IL-10 (JES5-2A5) antibody and rat IgG1 isotype control were purchased from BioXCell (USA). BMDCs were treated with 5 ng/ml of anti-mouse IL-10 or rat IgG1 isotype control for 2 h prior to DeinoLys and LPS treatment.

### Induction of Experimental Colitis

BALB/c mice (8-weeks-old) were housed in a pathogen-free environment. The mice were kept in an artificial 12 h light/dark cycle at 23°C–25°C and 45%–55% humidity and stabilized for 7 days. The mice were randomly divided into four groups: (1) normal, (2) dextran sulfate sodium (DSS) + PBS (control), (3) DSS + 75 mg/kg 5-aminosalicylic acid (5-ASA; positive control), and (4) DSS + 50 mg/kg DeinoLys (*n* = 6 per group). To induce colitis, 5% DSS (36–50 kDa, MP biomedical, Aurora, OH, USA) in drinking water was administered for 8 days. 5-ASA or DeinoLys was orally administered every 2 days from days 0 to 6 during treatment with DSS. All animal experiments were approved by the Institutional Animal Care and Use Committee of the Korea Atomic Energy Research Institute (KAERI-IACUC-2021-02).

### Evaluation of Colitis Symptoms

Body weight and disease activity index (DAI) scores were measured once every 2 days. The DAI score was calculated by combining the score of diarrhea (0 = normal, 2 = loose stool/loss of form, 3 = watery diarrhea, and 4 = no feces produced), bleeding (0 = no bleeding, 1 = visible blood in rectum, 2 = visible blood on the fur), and percentage of body weight loss (0 = lower than 1%, 1 = 1%–5%, 2 = 5%–10%, 3 = 11%–20%, and 4 = over 20% loss) following a previously established method [18]. On day 8, the mice were sacrificed by CO_2_ asphyxiation, and their colons were collected. For histological analysis, colon tissues were fixed with 10% buffered formalin phosphate (Sigma-Aldrich), embedded in paraffin, and stained with hematoxylin and eosin (H&E). Histopathological scores were measured by determining crypt distortion and lymphocyte infiltration according to the criteria published by Meer *et al*. [19].

### Measurement of Colonic Myeloperoxidase (MPO) Activity

The colon tissues were homogenized with radioimmunoprecipitation assay (RIPA) buffer (Invitrogen) using a homogenizer after being washed with cold PBS. The homogenized tissues were then centrifuged at 16,000 ×*g* for 20 min at 4°C, and the supernatant was collected. A bicinchoninic acid protein assay kit (Invitrogen) was used to determine the protein concentration in colon lysates, which was then normalized to 1 mg/ml. Following the manufacturer’s instruction, the colonic MPO level was determined using an MPO ELISA kit (Hycult BIotech, USA).

### Statistics

All of the experiments were repeated at least twice with triplicated wells. Statistical analysis was performed using one-way analysis of variance (ANOVA) followed by Tukey’s multiple comparison using GraphPad Prism 8.0 (GraphPad Prism Software, USA). The data in the graphs are expressed as means ± standard deviations (SD).

## Results

### DeinoLys Induces the Increase of IL-10 Production Simultaneously with Decrease of Proinflammatory Cytokines in LPS-Stimulated BMDCs

**Fig. 1:**
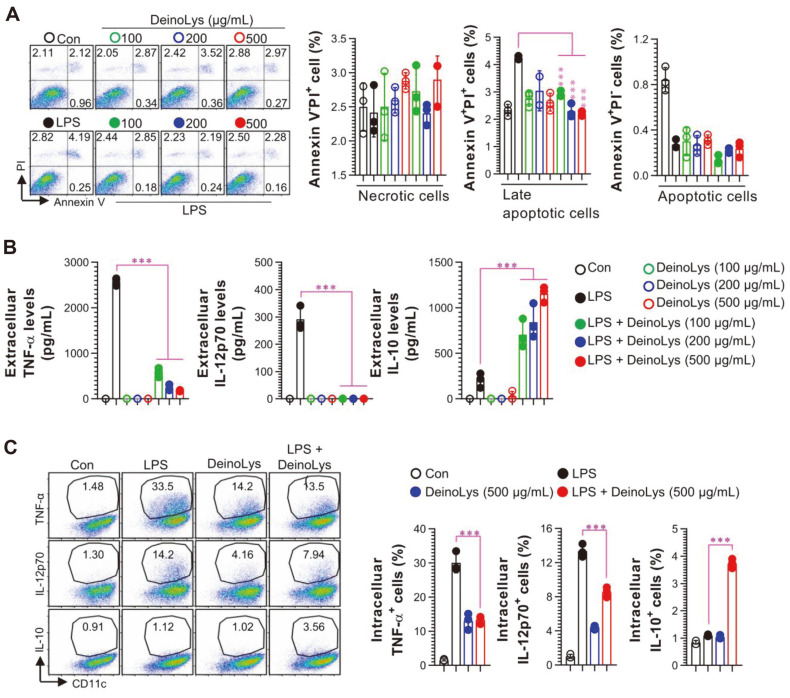
Effects of DeinoLys on cell viability and cytokine production in BMDCs. (**A**) Cells were treated with DeinoLys (100, 200, and 500 μg/ml) for 24 h in the presence or absence of LPS (100 ng/ml). In LPS + DeinoLys group, cells were co-treated with DeinoLys and LPS. Cell viability was analyzed by annexin V/PI staining. (**B**) Extracellular cytokine levels in the cell culture supernatant were measured using commercial ELISA kits. (**C**) Intracellular cytokine levels were determined by flow cytometry after 9 h in the presence of GoligPlug (1 μg/ml). The mean SD (*n* = 3) is shown in all bar graphs. One-way ANOVA was used for statistics, followed by Tukey's multiple comparison.***p < 0.001.

Prior to investigating the anti-inflammatory effects of DeinoLys in BMDCs, we first determined the noncytotoxic concentration of DeinoLys by annexin V/PI. We observed that DeinoLys did not exert cytotoxicity at concentrations up to 500 μg/ml in the presence or absence of LPS in BMDCs (Fig. 1A). Next, we determined whether DeinoLys could change the pattern of cytokine production in LPS-stimulated BMDCs. As shown in Fig. 1B, LPS-treated BMDCs exhibited increased levels of proinflammatory (TNF-α and IL-12p70) and anti-inflammatory (IL-10) cytokines in the cell culture supernatant. TNF-α and IL-12p70 production was significantly inhibited along with a remarkable increase in IL-10 levels in BMDCs cotreated with DeinoLys (100, 200, and 500 μg/ml) and LPS compared to the LPS only-treated group. The DeinoLys only-treated group showed no significant change in cytokine levels, as seen in Fig. 1B. The change in cytokine production by DeinoLys was confirmed at the intracellular level (Fig. 1C). The intracellular levels of TNF-α and IL-12p70 in the DeinoLys (500 μg/ml) and LPS cotreated groups were lower than those in the LPS only-treated group, whereas intracellular IL-10 level was increased. These results indicate that treatment with DeinoLys results in cytokine production with a tolerogenic phenotype of BMDCs in the presence of LPS stimulation.

### DeinoLys Decreases Surface Molecules in LPS-Stimulated BMDCs

**Fig. 2:**
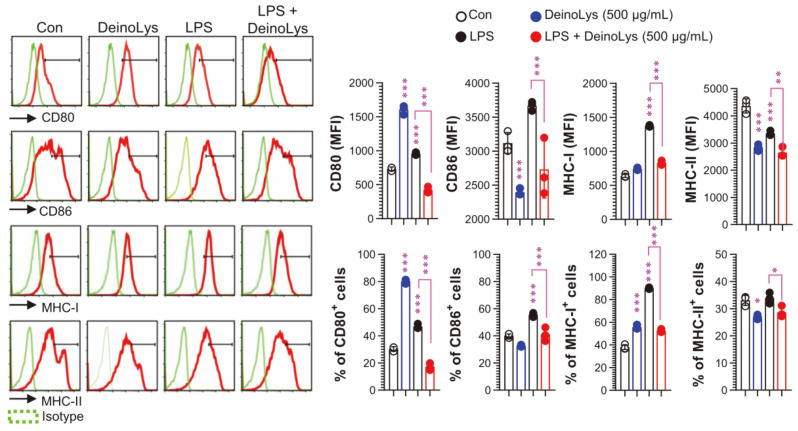
Effects of DeinoLys on surface molecules in BMDCs. Cells were treated with DeinoLys (500 μg/ml) for 24 h in the presence or absence of LPS (100 ng/ml). In LPS + DeinoLys group, cells were co-treated with DeinoLys and LPS. The cells were stained with each surface antibody (anti-CD80, anti-CD86, anti-MHC-I, and anti-MHC-II), pan-DC marker (anti- CD11c), live/dead kit, and analyzed by flow cytometry. Each surface moleculés mean fluorescence intensity in CD11c^+^ cells is represented by a bar graph. The mean SD (*n* = 3) is shown in all bar graphs. One-way ANOVA was used for statistics, followed by Tukey's multiple comparison.*p < 0.05, **p < 0.01, and ***p < 0.001.

We next determined whether DeinoLys could reduce the expression of surface molecules in BMDCs. Upon LPS stimulation, the expression levels of costimulatory molecules (CD80 and CD86), MHC-I, and MHC-II were significantly increased (Fig. 2). The DeinoLys only-treated DCs increased the expression level of CD80 with decrease of CD86 and MHC-II level. However, the expression of surface molecules was significantly decreased in the DeinoLys (500 μg/ml) and LPS cotreated group compared with the LPS only-treated group. These results indicate that DeinoLys inhibits LPS-induced phenotypic maturation of BMDCs.

### BMDCs Treated with DeinoLys in the Presence of LPS Stimulation Impair Allogenic T Cell Responses

**Fig. 3:**
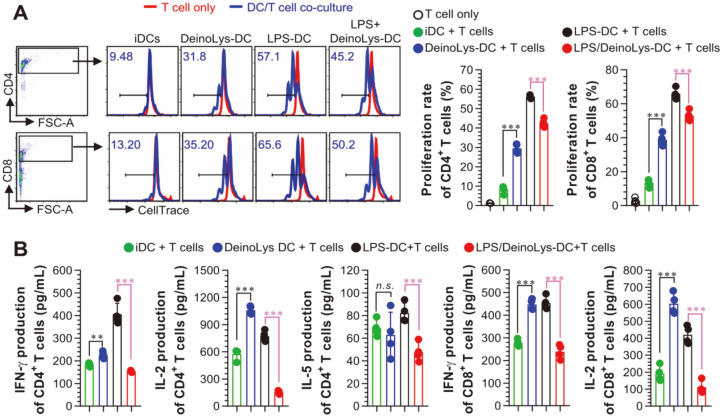
Effects of DeinoLys-treated BMDCs on proliferation and activation of T cells. (**A**) CD4^+^ or CD8^+^ T cells from BALB/c mice (allogenic mice) were prepared as described in the Materials and Methods. The cells were cocultured with DCs treated with PBS (iDC), DeinoLys, LPS, or LPS with DeinoLys for 48 h, and the proliferation of T cells was measured by flow cytometry. (**B**) Cytokine (IFN-γ, IL-2, and IL-5) levels in culture supernatant were measured using commercial ELISA kits. The mean SD (*n* = 3) is shown in all bar graphs. One-way ANOVA was used for statistics, followed by Tukey's multiple comparison.**p < 0.01 and ***p < 0.001.

DeinoLys-treated BMDCs displayed anti-inflammatory activity by changing to a tolerogenic phenotype in response to LPS stimulation, as illustrated above. Thus, we anticipated that BMDCs cotreated with DeinoLys and LPS would impair the proliferation and activation of allogenic T cell response. To verify this, we performed an MLR assay. The proliferation percentage of CD4^+^ or CD8^+^ T cells cocultured with DeinoLys only-treated or LPS only-treated BMDCs was higher than that of T cells cocultured with iDCs. Interestingly, the CD4^+^ or CD8^+^ T cells cocultured with LPS/DeinoLys-treated BMDCs showed a remarkably lower proliferation percentage than T cells cocultured with LPS only-treated BMDCs (Fig. 3A). Next, the levels of cytokines produced by activated T cells were measured in the culture supernatant. The levels of Th1- (IFN-γ and IL-2) and Th2-secreted (IL-5) cytokines in CD4^+^ T cells and Th1-secreted cytokines of CD8^+^ T cells were increased by the coculture of BMDCs stimulated with LPS. However, both Th1- and Th2-secreted cytokines were decreased in T cells cocultured with LPS/DeinoLys-treated BMDCs (Fig. 3B). Collectively, the results showed that proliferation/activation of CD4^+^ and CD8^+^ T cells cocultured with DeinoLys-treated BMDCs stimulated with LPS was effectively inhibited compared with T cells cocultured with LPS only-treated BMDCs. These findings indicate that DeinoLys impairs the interaction between DCs and T cells in the context of inflammation.

### IL-10 Neutralization Hinders Phenotypic Changes in Tolerogenic DCs by DeinoLys

**Fig. 4:**
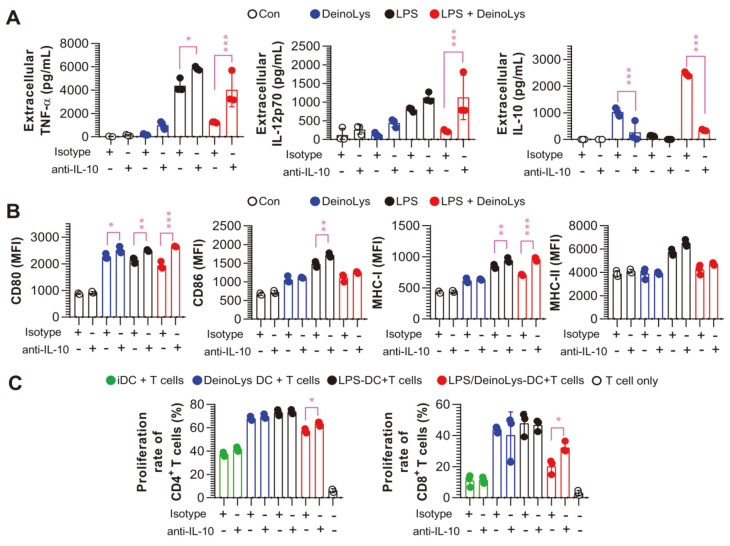
Effects of IL-10 neutralization on tolerogenic functions induced by LPS/DeinoLys-treated BMDCs. Cells were treated with anti-IL-10 mAb (5 ng/ml) or rat IgG (isotype control) for 2 h prior to LPS and DeinoLys treatment. (**A**) Extracellular cytokine levels in the cell culture supernatant were measured using commercial ELISA kits. (**B**) Surface molecules were measured by flow cytometry. The mean fluorescence intensity of each surface molecule in CD11c^+^ cells is represented by bar graphs. (**C**) CD4^+^ and CD8^+^ T cells isolated from splenocytes of BALB/c mice were stained with CellTrace™ Violet Cell Proliferation Kit and cocultured with DCs treated with PBS (Con), DeinoLys, LPS, or LPS with DeinoLys in the presence or absence of anti-IL-10 mAb or rat IgG. The proliferation of CD4^+^ or CD8^+^ T cells was analyzed by flow cytometry. The mean SD (*n* = 3) is shown in all bar graphs. One-way ANOVA was used for statistics, followed by Tukey's multiple comparison. *p < 0.05, **p < 0.01, and ***p < 0.001.

Based on the above findings, we hypothesized that IL-10 is a crucial factor in inducing tolerogenic DC phenotypes by DeinoLys in the presence of LPS. First, to investigate whether IL-10 production induced by DeinoLys in the presence of LPS significantly determines the tolerogenic phenotype of BMDCs, we added anti-IL-10 mAb or rat IgG (isotype control) antibody prior to LPS/DeinoLys treatment and then evaluated cytokine production and surface molecules. The inhibitory effects of TNF-α and IL-12p70 by DeinoLys in LPS-treated BMDCs were abrogated in the presence of anti-IL-10 mAb (Fig. 4A). Furthermore, the incubation of LPS/DeinoLys-treated BMDCs with an anti-IL-10 antibody restored the expression levels of CD80 and MHC-I compared with the rat IgG mAb-treated group (Fig. 4B). Next, we investigated the effect of IL-10 neutralization on the inhibition of allogenic T cell proliferation by LPS/DeinoLys-treated BMDCs under the same experimental conditions described in Fig. 3A. The proliferation percentage was restored in both CD4^+^ and CD8^+^ T cells cocultured with IL-10-neutralized BMDCs treated with LPS and DeinoLys compared with T cells cocultured with rat IgG-treated BMDCs (Fig. 4C). These results indicate that IL-10 is a key player in the tolerogenic phenotype of BMDCs following LPS/DeinoLys treatment.

### Oral Administration of DeinoLys Alleviates DSS-Induced Colitis Symptoms

**Fig. 5:**
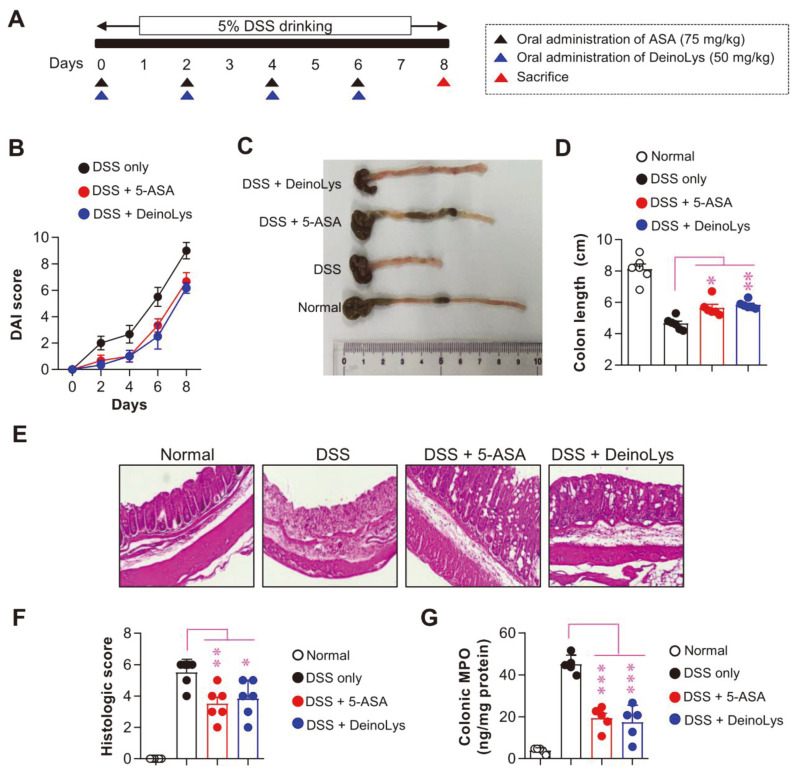
Effects of DeinoLys administration on DSS-induced colitis in mice. Mice drank water containing 5% DSS. 5-ASA (75 mg/kg, positive control) or DeinoLys (50 mg/kg/day) was orally administrated every 2 days from day 0 to 6 during treatment with DSS. (**A**) Experimental schedule for developing DSS-induced colitis symptoms and treating DeinoLys in BALB/ c mice. (**B**) DAI score was calculated by sum of diarrhea, bleeding, and loss of bodyweight score. (**C** and **D**) Mice were sacrificed on day 8, and the length of colons was measured. (**E** and **F**) Histological scores were analyzed using H&E-stained colon sections. (**G**) MPO enzymatic activity was measured to determine neutrophil infiltration into injured colon tissue. The mean SD (*n* = 6) is shown in all bar graphs. One-way ANOVA was used for statistics, followed by Tukey's multiple comparison. *p < 0.05, *p < 0.01, and ***p < 0.001.

Based on in vitro results, we hypothesized that DeinoLys could alleviate inflammatory symptoms in an ulcerative colitis mouse model. Thus, we evaluated the protective effects of DeinoLys against acute colitis symptoms using a mouse model that was orally administered 5% DSS. A schematic of experimental procedure is shown in Fig. 1A. Both the 5-ASA (positive control)-treated group (8 days; *p* < 0.05) and DeinoLys-treated group (6 days; *p* < 0.05, 8 days; *p* < 0.05) showed a lower DAI score than the control group (Fig. 5B). In addition, the DeinoLys-treated group showed a significantly longer colon than the DSS group (Figs. 5C and 5D). In histological analysis, 5-ASA-treated and DeinoLys-treated groups exhibited lower histological scores calculated by index of lymphocytes infiltration crypt loss than control group (Figs. 5E and 5F). Furthermore, the 5-ASA-treated and DeinoLys-treated groups showed reduced colonic MPO levels, which are considered markers of neutrophil infiltration (Fig. 5G). There is no significant change in bodyweight and colonic MPO level in between normal and DeinoLys only-treated group (Fig. S1).

## Discussion

In this study, the anti-inflammatory potential of DeinoLys (the hot water extract of *D. radiodurans* R1) as a pharmaceutical candidate for the treatment of IBD was explored.

The regulation of DC activation and maturation is considered a valuable therapeutic target in inflammatory disease therapy [20]. DCs recognize pathogen-associated molecular patterns, such as LPS, of the invading pathogens through membrane-bound pattern recognition receptors, which activate T cells by expressing high levels of costimulatory molecules and proinflammatory cytokines [21]. Conversely, tolerogenic DCs generally present antigens to T cells without costimulatory molecules, which leads to the inhibition of T cell proliferation due to insufficient IL-2 production [22, 23]. Furthermore, tolerogenic DCs produce high levels of anti-inflammatory cytokines, including IL-10 and transforming growth factor-β. Among them, IL-10, one of the most powerful anti-inflammatory cytokines, is a crucial soluble molecule involved in the initiation of homeostatic and anti-inflammatory programs [24]. Excessive IL-10 production can inhibit tyrosine phosphorylation of CD28, which enables T cell growth inhibition and IFN-γ and IL-2 release [25]. In addition, IL-10 producing DCs can cause anergic T cell responses and suppress activated Th1 and Th2 responses [26]. For this reason, the induction of IL-10 producing tolerogenic DC is considered an effective therapeutic strategy for inflammatory diseases [24]. As shown in Figs. 1–3, we observed that DeinoLys treated BMDCs exhibit a tolerogenic phenotype in the presence of LPS stimulation, resulting in the inhibition of CD4^+^ and CD8^+^ T cell activation and proliferation. The anti-inflammatory effects of DeinoLys were abrogated by IL-10 neutralization (Fig. 4). Interestingly, the increased level of surface molecules in DeinoLys-only treated DCs and the activated T cells co-cultured with DeinoLys-only treated DCs were observed; conversely, these properties of the activated DCs by DeinoLys were changed into tolerogenic DCs in the presence of TLR4 stimulation by LPS. Similarly, despite the fact that apathogenic Gram-negative commensal bacteria, *Bacteroides vulgatus* induces semi-maturation of DCs, *B. vulgatus*-treated DCs with TLR stimulation by *E. coli* exhibited the anti-inflammatory properties [27]. Furthermore, when TLR2 was involved, *Vitreoscilla filiformis* lysates, a non-pathogenic bacteria, induced IL-10-producing DCs, effectively alleviating skin inflammation [28]. In accordance with previous studies, DeinoLys could induce anti-inflammatory responses by switching DC function and phenotype into IL-10-producing tolerogenic DCs, but TLR4 signal is required. We speculated that DC maturation by DeinoLys in the absence of TLR4 stimulation was due to activation of other immune pathways. Further studies will be required to determine whether DeinoLys can modulate other parts of the immune system.

DSS, a polymer of sulfated polysaccharide, induces mucosal injury and inflammation caused by direct hyperosmotic damage to epithelial cells [29]. The repeated administration of DSS causes diarrhea, bleeding, mononuclear leukocyte infiltration, and crypt loss, which is widely used as an experimental colitis model because of its reproducibility and similarities with human ulcerative colitis [30, 31]. Previous studies have demonstrated that treatment with bioactive substances inducing a tolerogenic phenotype of DCs effectively attenuated colitis symptoms [32-34]. In particular, IL-10 plays an important role in the protection and delay of disease progression in intestinal inflammation [35]. Importantly, IL-10 helps to reduce inflammation indicators like MPO, which is a marker for active neutrophil infiltration [36]. Thus, we examined whether oral administration of DeinoLys alleviates colitis symptoms using a DSS-treated mouse model. Our data showed that oral administration of DeinoLys attenuated colitis clinical signs, histopathological changes, and colonic MPO level in a DSS-induced colitis mouse model (Fig. 5).

In conclusion, our results suggest that DeinoLys regulates the phenotype and function of BMDCs into tolerogenicity via IL-10 production, which is capable of inhibiting T cell proliferation and activation. In a colitis mouse model, DeinoLys treatment attenuates DSS-induced colitis symptoms. These results indicate DeinoLys as a potential therapeutic candidate for ulcerative colitis. These findings will lead to more diverse and advanced utilization of *D. radioduran*-derived components in the field of medicine. However, more research evaluating the components of DeinoLys is needed for its use as an anti-inflammatory candidate.

## Supplemental Materials

Supplementary data for this paper are available on-line only at http://jmb.or.kr.
