# Supplementary material for: Deinococcus radiodurans R1 Lysate Induces Tolerogenic Maturation in Lipopolysaccharide-Stimulated Dendritic Cells and Protects Dextran Sulfate Sodium-Induced Colitis in Mice
Source: J Microbiol Biotechnol. 2022 Jun 7;32(7):835–43. doi: 10.4014/jmb.2203.03008 (PMC9628914; doi:10.4014/jmb.2203.03008)
Supplement: Supplementary file 1 [file jmb-32-7-835-supple.pdf]

J. Microbiol. Biotechnol.
https://doi.org/10.4014/jmb.2203.03008

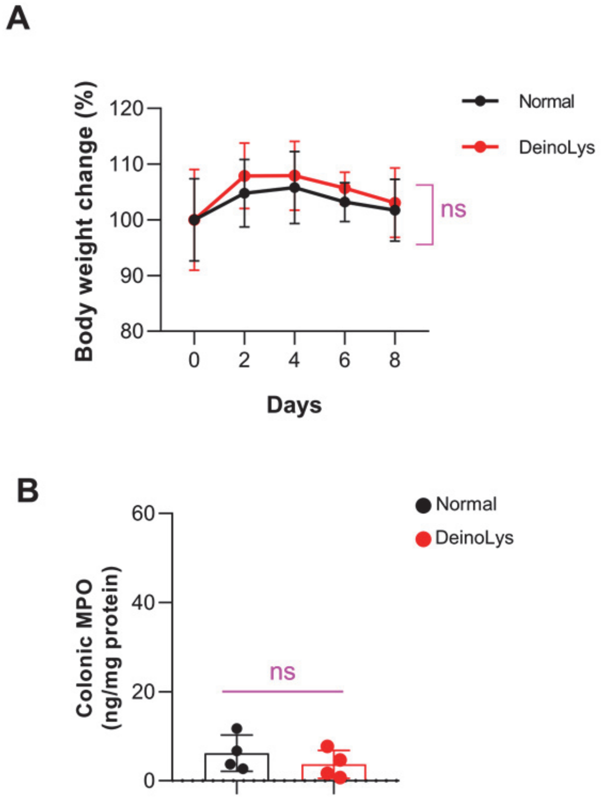

Supplemental Fig. 1. Effects of DeinoLys administration on normal mice

DeinoLys (50 mg/kg) was orally administrated every 2 days without treatment of DSS. (A) For 8 days, bodyweight of mice was measured every 2 days. (B) Mice were sacrificed on day 8, and colon tissue was homogenized with RIPA buffer using homogenzier. The colonic MPO level was measured using MPO ELISA kits. The mean SD (*n* = 4) is shown in all bar graphs. Unpaired Students' t test was used for statistics.
